# Individual differences in parasympathetic nervous system reactivity in response to everyday stress are associated with momentary emotional exhaustion

**DOI:** 10.1038/s41598-024-74873-9

**Published:** 2024-11-04

**Authors:** Regina Franziska Schmid, Joachim Thomas, Katrin Rentzsch

**Affiliations:** Department of Psychology, Catholic University of Eichstätt-Ingolstadt, Eichstätt, Germany

**Keywords:** Physiological stress reactivity, Heart rate variability, Perceived time pressure, Emotional exhaustion, Ecological momentary assessment, Physiology, Psychology

## Abstract

Acute stressors (e.g., time pressure) can provoke psychological and physiological stress responses, and the magnitude of such responses is called stress reactivity. However, stress reactivity levels can differ considerably among individuals, with exaggerated levels being associated with adverse outcomes (e.g., emotional exhaustion). Previous studies have primarily investigated psychological stress reactivity or physiological stress reactivity induced in the laboratory. Physiological stress reactivity, especially concerning heart rate variability (HRV), has rarely been examined so far in real life. We addressed this research gap in a sample of 394 adults who participated in 2- to 4-day ecological momentary assessments. Individuals answered self-reports on perceived time pressure and emotional exhaustion multiple times a day and simultaneously wore electrocardiogram sensors. Based on 4,009 total situations and 3–16 situations per participant, individual differences in HRV reactivity to time pressure were computed as random slopes from multilevel models. Consistent with preregistered hypotheses, increased time pressure was associated with reduced HRV, and increased stress reactivity was associated with increased emotional exhaustion. The findings highlight the detrimental effects of everyday demands and physiological reactivity and emphasize the relevance of practical coping strategies. This study contributes to research on dynamic inter- and intraindividual stress regulation using ambulatory, psychophysiological methods.

## Introduction

Emotional exhaustion is a core dimension of burnout and a negative indicator of psychological well-being. It can negatively impact mental and physical health, impair functioning, and reduce life satisfaction^1,2^. One important factor that influences emotional exhaustion is the way people respond to a stressful situation in daily life, and individual responses to it are regarded as more decisive than the situation itself^3^. In line with many theoretical perspectives, it has been argued that *physiological *stress reactivity in particular (i.e., objectively measurable bodily changes in response to stressors) is associated with emotional exhaustion^4,5^, yet most empirical research has been limited to *psychological *stress reactivity (i.e., subjective, self-reported emotional or affective changes in response to stressors)^6^. The few studies that have investigated outcomes of physiological reactivity have primarily been conducted in laboratory settings^7^. However, physiological reactivity outcomes in response to everyday stressors have rarely been explored. The present study aims to close this research gap by implementing a novel and ecologically valid approach in which real-life heart rate variability (HRV) reactivity is examined as individual differences in within-person stressor-strain relationships derived from ecological momentary assessments (EMAs).

### Psychophysiological stress responses

Being confronted with acute stressors in everyday life evokes emotional, behavioral, and physiological changes in an individual’s organism. In recent decades, prominent models, such as the allostatic load model^5^, the transactional model of stress^8^, and the job demands-resources model^1^, have provided theoretical background for the prediction of psychophysiological strain in response to stressors. In the present study, we focused on acute (and not chronic) stressors, such as time pressure, as well as on short-term (and not long-term) stress responses, such as temporary adaptations in physiology. Reviews of laboratory-based evidence have shown that such physiological stress responses come along with changes in the sympatho-adrenal medullary (SAM) axis (e.g., involving an increase in heart rate) and in the hypothalamic-pituitary-adrenal (HPA) axis (e.g., involving a release of cortisol^9,10^). Similarly, real-life studies with mobile physiological measurements have linked situational demands with phasically increased ambulatory heart rate and blood pressure as well as reduced HRV^11,12^. However, not every stressor evokes stress-related responses in all individuals equally. The magnitude of the stress response can therefore vary considerably from individual to individual and is often defined as *stress reactivity*^13–15^.

While moderate and temporary hormonal, cardiovascular, and inflammatory stress reactivity are considered adaptive, as they facilitate the body’s ability to cope with stress, an increased and sustained stress reactivity is associated with biological wear and tear^5^. When an organism experiences exaggerated stress reactivity, the organism unnecessarily mobilizes energy to prepare for action, which is not considered appropriate for modern psychosocial stressors because—in contrast to ancient physical, life-threatening stressors—modern stressors do not require a fight-or-flight response^16^. According to the reactivity hypothesis and stress reactivity theory, these “metabolically unjustified responses”^10^ (p. 2) imply a loss of homeostatic regulation, which may unfold health-impairing long-term effects via either overactivation of the SAM and HPA axes or stress-evoked unfavorable health behaviors such as maladaptive coping^17^. Previous research has consistently shown that stronger responsiveness to stress is linked to global perceived distress and negative affectivity^3,15,18,19^, and it prospectively predicts mental and physical diseases as well as mortality^10,17,20,21^.

### Measuring stress reactivity with psychological and physiological instruments

There is an ongoing debate about how stress reactivity can best be measured and operationalized^22^. Previous research has focused primarily on self-reported psychological stress reactivity. For example, Schulz and colleagues^15 ^developed a stress reactivity scale that assesses the perceived typical duration and magnitude of affective reactions to different stressors, from which a sum score that represents an individual’s stress reactivity can be calculated^14,15^. Although self-report questionnaires have many advantages, they lack the ability to measure stress responses in an ecologically valid way because they survey only typically perceived response patterns and do not include situational information about the current moment. Other approaches have operationalized psychological stress reactivity by comparing self-reported affect or mood in stressful versus neutral conditions. In this sense, stress reactivity is typically quantified via difference scores in the mean outcome values^23^. However, psychological measures of stress reactivity are limited due to their susceptibility to socially desirable or otherwise biased responses. In addition, self-reports can rely only on consciously accessible information, whereas physiological measures can also be indicative of unconscious processes^4,24^.

To objectively measure stress reactivity, the use of physiological parameters can be advantageous, as they reduce many of the described limitations associated with self-reports. Among the broad variety of SAM and HPA biomarkers, HRV has been established as a prominent and increasingly used metric in stress-related literature^9,25^. HRV refers to millisecond variations in time intervals of consecutive heartbeats (NN intervals) derived from an electrocardiogram (ECG) and is primarily driven by the aforementioned SAM axis, covering both sympathetic and parasympathetic (vagal) branches of the autonomic nervous system^26^. Often quantified by the root mean square of successive NN differences (RMSSD), which represents vagally mediated activity, reductions in HRV have been widely associated with experimental stress provocation in laboratory settings^23,27,28 ^but also with momentary stressful, unpleasant events or worry episodes in real life^11,25,29,30^. In addition to these stressor-strain relationships, HRV has in fact also been previously used to compute physiological stress reactivity in the laboratory, linking this measure to emotional disorders^31 ^or poor decision-making^7^.

### Measuring stress reactivity in laboratory and real-life settings

For decades, researchers have used laboratory tasks to study psychological^27 ^or physiological^28 ^responsiveness to stress. In so-called rest-reactivity-recovery designs, the trier social stress test (TSST) is one of the most frequently used research paradigms for inducing stress and measuring stress reactivity^32^. It consists of social-evaluative and unpredictable stress tasks usually preceded by a baseline condition at rest. In a recent review, Allen and colleagues^33^ linked responsiveness to the TSST to a multitude of stress indicators, including cortisol as well as immune, autonomic, and cardiovascular parameters.

Although laboratory-based designs have been informative in terms of measurement precision, they come with the limitation that they are not particularly ecologically valid, nor do they usually account for within-person variations^24^. Recent accounts on laboratory-based virtual reality environments^34 ^can be appreciated as a step in the right direction, as they combine experimental control and authentic job demands; nonetheless, they stick to laboratory conditions, and previous studies have indicated that findings on responsiveness to laboratory stress cannot be generalized to naturalistic environments^23,35^. Accordingly, scholars have called for analyses of reactivity patterns in everyday circumstances to gain insights into such processes in real life^36 ^and to prevent biases that come with laboratory settings (e.g., white coat hypertension effect^37^).

A similarly critical view^29,36 ^has been directed toward the predominant between-person perspective, with studies commonly characterizing stress reactivity as a static and fixed measure that relies on single stressors and aggregated response values. Sliwinski and colleagues^38^, for instance, argued that “reactivity varies reliably not only between individuals but also within individuals across time” and that it changes “rapidly across brief time periods” (p. 830). It is crucial to address such micro-level mechanisms in stress reactivity research, particularly because this level best reflects people’s actual reactivity.

Researchers have attempted to remedy these shortcomings associated with laboratory-based research by conducting EMA studies, which are regarded as particularly ecologically valid^39^. This method typically relies on smartphone-based self-reports answered several times per day, for example, about current stressors and experiences, which can be combined with wearable devices that take physiological measurements^40^. By repeatedly or continuously collecting data while people pursue their normal daily routines, both inter- and intraindividual fluctuations can be observed in real-life, natural contexts^24^.

### Stress reactivity defined as individual differences in within-person associations

Building on the framework of the whole trait theory^41^, individual differences (e.g., in stress reactivity) may best be characterized by between-person differences in intraindividual, situation-state contingencies^18,42^. For example, Timmons and colleagues^36 ^calculated real-life physiological reactivity slopes on the basis of hourly feelings of annoyance and responses in electrodermal activity (EDA) and examined them in relation to interpersonal aggression. A handful of EMA studies have already drawn on this idea in stress reactivity research and have defined stress reactivity as between-person differences in the strength of within-person relationships between daily stressors and stress responses^3,17,21^. These studies have incorporated such within-subjects slopes of daily stressor-strain relationships and subsequently modeled these reactivity coefficients as predictors of other outcomes^18^. For example, Charles and colleagues^3^ conducted a daily diary study on daily stressors and self-reported negative affect and quantified affective stress reactivity “as a slope representing the difference in levels of negative affect on days when a stressor occurred compared with days when no stressors occurred” (p. 735). Still, these studies have concentrated solely on psychological stress reactivity, while mechanisms of naturalistic physiological reactivity are not yet sufficiently understood. To conclude, within the scope of stress research, there is still a need for an operationalization of physiological, and specifically HRV, reactivity as a within-person slope covering multiple assessments in daily life and the investigation of its effects on acute everyday well-being.

### The present research

In the present research, we investigated the relationship between situational physiological stress reactivity and momentary psychological well-being from a real-life perspective using EMA. We combined repeatedly answered self-reports of current stressors (i.e., perceived time pressure) and well-being (i.e., emotional exhaustion) with continuously recorded ECG measurements, which we used for the HRV analysis. Bearing in mind the predominant between-person research focus on psychological stress reactivity or physiological stress reactivity induced in the laboratory, the present study aimed to extend real-life physiological stress reactivity research by examining naturalistic, inter- and intraindividual dynamics of the under-researched HRV reactivity. Specifically, we investigated whether amplified short-term HRV reactivity to momentary perceived time pressure is associated with increased situational emotional exhaustion in daily life.

The following hypotheses were preregistered prior to data analysis (see https://osf.io/jxd93). Based on previous findings on the relationship between acute demands and vagal tone^11^, we expected a negative, significant association between situationally perceived time pressure and state HRV. From this multilevel model, in turn, we extracted person-specific slopes representing the amount of change in HRV in the context of stressors. We thereby provide a unique operationalization of physiological reactivity, as it models interindividual tendencies in stress reactivity on the basis of repetitions of short-term stress processes. In doing so, we were able to capture fine-grained dynamics that occur during stress reactions in everyday life better than traditional (i.e., laboratory-based, one-time) approaches have been able to do^18^. Building on previous evidence on associations between physiological reactivity and well-being^16^, we hypothesized that individuals showing greater parasympathetic nervous system reactivity in response to time pressure are more likely to experience higher levels of emotional exhaustion in everyday life.

## Methods

### Participants

The total sample consisted of *N*_*2*_ = 394 (129 male; 263 female; 2 non-binary) adults in southern Germany pooled together from five subsamples (*n*_*2*_ = 68 students with *n*_*1*_ = 989 situations, *n*_*2*_ = 78 police officers in training with *n*_*1*_ = 792 situations, *n*_*2*_ = 101 teachers with *n*_*1*_ = 1,193 situations, *n*_*2*_ = 88 healthcare professionals with *n*_*1*_ = 1,090 situations, and *n*_*2*_ = 59 managers in nursing care with *n*_*1*_= 577 situations) collected between 2018 and 2023. Data from two subsamples (i.e., teachers and healthcare professionals) have previously been published^40,43^, albeit in relation to other research questions and with other variables that do not play a role here (see the preregistration for further details).

Initially, 480 individuals registered to participate in the studies. Due to sick leave and dropout (*n*_*2*_ = 30), insufficient or missing HRV recordings (*n*_*2*_ = 23), technical failure (*n*_*2*_ = 10), and excessive artifacts (*n*_*2*_ = 23), 86 participants needed to be excluded on the basis of preregistered exclusion criteria. The participants in the final sample were on average 34.8 years old (*SD* = 14.3) and had a mean body mass index (BMI) of 24.18 kg/m^2^ (*SD* = 4.24). The following inclusion criteria for participating in the studies applied to all subsamples except the student sample: no heavy smoking or alcohol consumption, no excessive physical activity, and no cardiovascular, mental, or metabolic diseases or related medication. The student sample differed in that it included *n*_*2*_ = 3 smokers and *n*_*2*_ = 10 individuals with cardiovascular, mental, or metabolic diseases or medications; however, the visual inspection of the ECG recordings revealed no differences between these individuals and the remaining healthy, non-smoking sample; therefore, they were included in the analysis. All participants signed an informed consent form and received individual feedback on HRV parameters.

Regarding statistical power, the recommended minimum number for estimating variances and covariances of random effects in multilevel modeling is *N*_*2*_ = 100 for level 2 (L2) units with *n*_*1*_= 10 level 1 (L1) measurements per unit^44^. However, we aimed for a larger sample than the recommended minimum, first, because we wanted to compensate for the exclusion of individuals and, second, because we wanted to robustly estimate random slopes and their relationships with other variables^42,45^.

The ethics committee of the Catholic University of Eichstätt-Ingolstadt gave approval for the data to be collected (approval numbers: 2018/01, 2019/09, 018–20, 111–2022, 125–2022).

### Design and procedure

The EMA period lasted for 2–4 days in all subsamples and combined continuous physiological recordings throughout the entire measurement period with repetitive smartphone-based self-reports occurring at fixed or semi-random times for each subject. Please note that the sampling days cover leisure time as well as studying or working hours and, to a much lesser extent, night shifts. Participants were recruited via various channels: oral presentations, flyers, posters, and e-mail lists. Interested individuals were given detailed written information and could register for the study. Before data collection began, participants were provided with a study package that was anonymized via a participant code and contained a smartphone, the ECG equipment, and a charger as well as further instructions. All materials were returned after participation.

### Measures

#### Self-reports during the ecological momentary assessment procedure

Self-reports were collected via questionnaires presented via the movisensXS application (movisens GmbH, Karlsruhe, Germany) on each smartphone. Fixed or semi-random sampling schemes were used. Participants received 4–6 notifications per day. The time windows for the notifications differed across subsamples, but all notifications were delivered between 7:30 AM and 9:00 PM, except for the subsample of healthcare professionals who were partly also notified during night shifts (for more details, see^43^). The minimum time between notifications was one hour. Following the initial notification, the EMA questionnaire was available to the participant for up to 45 minutes. Once the questionnaire had been opened, it timed out if the participant had not completed an individual item within 90 seconds. All items were answered on seven-point rating scales (1 = “strongly disagree”; 7 = “strongly agree”) and were treated as metric, linear measures based on the assumption of equal intervals between responses on the scale.

*Time pressure. *Time pressure was measured by adapting an item from the German version^46 ^of the Copenhagen Psychosocial Questionnaire^47^: “…I was under time pressure.” Note that the introductory sentences differed slightly between the subsamples (e.g., “In the last two hours...”; “In the last few hours...”; “In the last hour...”).

*Emotional exhaustion. *The outcome variable emotional exhaustion was assessed with the single-item “At the moment, I feel emotionally exhausted”. The item is in accordance with the concept of emotional exhaustion as it is addressed in various established burnout questionnaires such as the Copenhagen Burnout Inventory^48 ^or the Maslach Burnout Inventory^2^.

#### Physiological recordings using ambulatory assessment and data processing

Physiological data were collected continuously throughout the entire measurement period using single channel EcgMove3 and EcgMove4 sensors attached to a chest strap with two dry electrodes (movisens GmbH, Karlsruhe, Germany). The device recorded ECG signals with a sampling rate of 1,024 Hz and bodily movement with 64 Hz. After prior instructions, all participants applied the chest strap on their own at the height of the sternum. We used the Movisens DataAnalyzer to further process the ECG and movement data (movisens GmbH, Karlsruhe, Germany). The procedures and algorithms associated with artifact detection, R-peak detection, NN-List generation, segmentation, segment validation, and detrending are thoroughly documented on the manufacturer’s website (https://docs.movisens.com/Algorithms/ecg_hr_hrv/#r-peaks-r). State HRV and state bodily movement were operationalized as the 5-minute means of RMSSD and three-dimensional movement acceleration, respectively, preceding the EMA questionnaire. This duration was defined in accordance with established guidelines^26^. We chose RMSSD because this HRV parameter has been shown to reliably reflect short-term vagus activity in ambulatory studies^49^. To reduce skewness, we transformed the parameter with a natural logarithm (lnRMSSD). Prior to data analysis, in line with preregistered criteria, the ECG material was visually inspected for excessive artifacts, extraordinary beats, and ventricular extrasystoles to identify and exclude these individuals from the analyses (see the Participants section for the exact numbers of excluded participants).

### Analytic strategy

We analyzed the data in accordance with our preregistered analytical protocol. Due to the two-level data structure with repeated measurements (L1) nested within individuals (L2), we addressed the present hypotheses by using random-coefficient multilevel modeling^50^. Linear mixed-effects models were computed with the lme4-package in R^51^, following the analytical strategy by Wagner and colleagues^42^. To investigate our first hypothesis about the relationship between momentary perceived time pressure (L1 predictor) and state HRV (L1 outcome), we applied a random intercept and slope model while controlling for momentary bodily movement (L1 covariate). The L1 predictor (perceived time pressure) and covariate (bodily movement) were person-mean centered prior to running the analyses^39^. To prepare to test the second hypothesis on the link between stress reactivity and emotional exhaustion, we extracted the random slope coefficients from the model described above, which indicate between-person differences in physiological HRV reactivity to the momentary demands of perceived time pressure. These person-specific coefficients were entered as L2 predictors of situational emotional exhaustion (L1 outcome) in a means as outcome model. The Table contains the final model estimates and chi-square tests for quantifying model fit based on log likelihood ratios. We conducted two-tailed tests using the inference criterium *p* < .05. To increase transparency and reproducibility in research, the codebook, the anonymized data, and the R code are available on the Open Science Framework (OSF; https://osf.io/gwxj3).

## Results

### Descriptive analyses

A total of 4,641 smartphone prompts were sent to the participants, of which 4,009 were answered, 472 were ignored, and 112 were dismissed, corresponding to a compliance rate of 87%. Out of a maximum possible 16 smartphone questionnaires, each person responded to an average of 10.30 questionnaires (*SD* = 2.73, range: 3–16). Table 1 shows mean scores, standard deviations, intraclass correlation coefficients (*ICC*s), and within- and between-subject correlations for all study variables. The *ICC*s showed that 52% of the variance in state HRV was located at the between-person level, as was 45% of the variance in emotional exhaustion, 37% in time pressure, and 22% in bodily movement. These findings mean that all study variables exhibited substantial variation both within and between individuals, underscoring the appropriateness of multilevel analyses.

**Table 1:** Descriptive statistics and correlations between variables.

	Variable	*M*	*SD*_w_	*SD*_b_	*ICC*	1	2	3	4
1	HRV (lnRMSSD)	3.28	0.66	0.48	.52	--	–.45***	–.12***	–.04**
2	Bodily movement	0.07	0.26	0.03	.22	–.06	--	.14***	.02
3	Time pressure	2.93	1.25	1.07	.37	–.17***	.01	--	.14***
4	Emotional exhaustion	2.53	1.14	1.13	.45	–.09	–.11*	.42***	--
5	Physiological stress reactivity	–0.02		0.01		–.13*	–.13**	.10	.10*
Note. *N*_*2*_ (persons) = 394, *N*_*1*_ (assessments) = 4,009. *SD*_w_ = within-person standard deviation. *SD*_b_ = between-person standard deviation. *ICC* = intraclass correlation coefficient. Correlations above the diagonal are level 1 correlations, and those below the diagonal are level 2 correlations that rely on aggregated data. HRV = heart rate variability. lnRMSSD = log-transformed root mean square of successive differences. * *p* < .05. ** *p* < .01. *** *p* < .001.									

### Physiological stress reactivity: effect of time pressure on heart rate variability

To address Research Question 1, we investigated whether perceived time pressure was related to reduced HRV. While controlling for momentary bodily movement (γ = −2.91, *t*(239) = −17.12, *p* < .001), we found that situationally perceived time pressure (γ = −0.02, *t*(211) = −4.01, *p* < .001) significantly predicted diminished levels of state HRV (see Table 2). This finding confirmed Hypothesis 1 on the stressor-strain relationship, meaning that, in situations in which participants experienced more time pressure, they showed temporarily reduced HRV, indexing a typical physiological stress response when confronted with acute stressors.

**Table 2:** Multilevel models predicting heart rate variability (stress reactivity model) and emotional exhaustion.

	HRV (lnRMSSD)				Emotional exhaustion			
**Fixed effects**	Estimate (*SE*)	*df*	*t*	*p*	Estimate (*SE*)	*df*	*t*	*p*
Intercept	3.29 (0.02)	393	135.00	< .001***	2.72 (0.11)	392	24.58	< .001***
Bodily movement	–2.91 (0.17)	239	–17.12	< .001***				
Time pressure	–0.02 (0.00)	211	–4.01	< .001***				
Physiological stress reactivity					10.22 (5.18)	390	1.97	.049*

**Random effects variances**	Estimate (*SD*)				Estimate (*SD*)			
Variance intercept	0.22 (0.47)				1.10 (1.05)			
Variance bodily movement	4.27 (2.07)							
Variance time pressure	0.00 (0.03)							
Residual variance	0.12 (0.35)				1.67 (1.29)			

**Model comparison**	Null model	Covariates model	Main effects model		Null model		Main effects model	
–2* Log likelihood	5910.60	4844.91	4115.57		14241.29		14232.28	
Δ–2*Log likelihood		1065.69***	729.34***				9.01**	
*df*		3	4				1	

Note. *N*_*2*_ (persons) = 394, *N*_*1*_ (situations) = 4,009. lnRMSSD = log-transformed root mean square of successive differences.

* *p* < .05. ** *p* < .01. *** *p* < .001.

### Effect of physiological stress reactivity on emotional exhaustion

With regard to Hypothesis 2, we expected a positive association between stronger HRV reactivity to perceived time pressure and momentary emotional exhaustion. By extracting the random slope coefficients from the previous model that reflected physiological reactivity in everyday life and entering them into the current model, we found that stronger HRV reactivity was significantly and positively related to momentary emotional exhaustion (γ = 10.22, *t*(390) = 1.97, *p* = .049). Consistent with our hypothesis, individuals with elevated levels of physiological reactivity were on average more emotionally exhausted in daily life than participants whose cardiovascular reactivity to time pressure was less pronounced.

## Discussion

With the present study, we aimed to gain deeper insights into physiological stress reactivity patterns while people performed their routine activities. In doing so, we applied a multimethodological EMA approach in which multiple smartphone questionnaires were combined with continuous ECG recordings. Using a heterogenous sample of employees, trainees, and students, the current study analyzed whether individual differences in vagally mediated reactivity to time pressure were associated with elevated emotional exhaustion in everyday life. In doing so, we addressed two major avenues for future stress research, that is, investigating daily stress reactivity under natural real-world (as opposed to laboratory) conditions and placing a stronger focus on physiological (as opposed to psychological) indicators^6^. Additionally, we built on current endeavors in differential psychology research by operationalizing interindividual tendencies (e.g., in stress reactivity) on the basis of repeated situation-state observations rather than one-time measurements^18^. In line with previous work^11,16^, our preregistered hypotheses were fully supported such that intraindividually increased time pressure was linked to temporarily reduced HRV, and interindividually increased HRV reactivity to time pressure was linked to higher mean levels of emotional exhaustion. Therefore, the present research adds to the literature by leveraging methodological and statistical advancements in operationalizing stress reactivity and elucidating the effects of real-life physiological stress reactivity on momentary emotional well-being.

Supporting Hypothesis 1 on the stressor-strain relationship, the current study found that situations that were perceived as under time pressure were accompanied by intermittent reductions in HRV. That is, while accounting for momentary bodily movement, there were acute physiological changes when people were pressed for time, reflecting parasympathetic withdrawal in response to stress. This result matches the classical fight-or-flight response, which prepares the body to meet environmental demands^52^, and is consistent with theories postulating that stressful demands are associated with psychophysiological strain reactions^1,5,8^. The finding also replicated previous evidence showing demand-induced fluctuations in HRV, in both laboratory^23,27 ^and real-life settings^11,25^. Future research dedicated to explain additional between-person variations by adding individual difference variables such as neuroticism or resilience could shed further light on how this psychophysiological stressor-strain-relationship varies between people. Notably, other ambulatory studies have also revealed opposite or nonsignificant relationships between acute stressors and HRV^40,53^, which may have indicated that the magnitude of stress responses differs considerably between individuals, creating a bridge to our second hypothesis.

In line with Hypothesis 2 on the detrimental effect of physiological stress reactivity on emotional well-being, we found a significant positive relationship between interindividual HRV reactivity (operationalized as within-subject slopes from the previous model) and momentary emotional exhaustion. That is, individuals exhibiting greater HRV reactivity to time pressure were on average more likely to be emotionally exhausted in daily life. These findings comply with those reported in studies of psychological reactivity^3,18 ^and in studies of laboratory-induced physiological reactivity^10,31^. The present result also converges with resource-oriented models of well-being^54,55 ^that theorize that the more physiological and energetic resources are mobilized and invested in stress coping, the more likely an individual will be to suffer from losses in emotional well-being. Similarly, our findings are in line with psychophysiological HRV theories^52,56,57 ^that argue that people with lower HRV reactivity have more resources available to meet the self-regulatory demands of stressors and are thus more likely to maintain their well-being^58^.

Our findings have essential theoretical and methodological implications. First, we enriched previous research by confirming relationships with real-life physiological stress reactivity, which had previously primarily been studied with psychological or laboratory-based physiological stress reactivity. By shedding light on how daily stressors, parasympathetic nervous system reactivity, and emotional well-being are intertwined, our study fosters a more holistic understanding of psychophysiological regulatory mechanisms. Next, our findings suggest that it is worth incorporating approaches from differential psychology (e.g., whole trait theory^41^) into existing theories of physiological stress reactivity^5^. By integrating findings from different disciplines, the present study contributes to a better understanding of inter- and intraindividual differences in shaping physiological stress reactivity and emotional well-being in daily life. Thus, the present research calls for stress researchers to see stress reactivity under a new lens that explicitly acknowledges real-life, within-person, multimodal perspectives.

From a methodological point of view, the operationalization of physiological HRV stress reactivity as person-specific random slopes obtained from multilevel models also goes beyond the scope of previous stress reactivity research^18^. We assessed time pressure and state HRV multiple times a day across several days, covering a multitude of situations with different contextual characteristics. Since stress and reactivity processes are very fast-moving, the EMA approach with its frequent measurements is a particularly suitable method. Therefore, we were able to capture the dynamics as they occurred in daily life quite closely and to describe interindividual differences in people’s typical reactions to stress in a reliable and ecologically valid way. Finally, our study benefits from a reduced risk of common method variance, as we used two different (i.e., self-reported and physiological) data collection techniques. This approach increases the amount of “true” shared variance and prevents measurement errors as well as spurious, inflated correlations, thereby enhancing the validity of our conclusions^59^.

In terms of practical implications, ecologically valid diagnostics accounting for intraindividual dynamics and interindividual differences is essential for identifying risk groups in which reduced well-being must be prevented through practical coping and recovery strategies. On the basis of our finding that physiological stress reactivity is associated with emotional well-being, the question arises as to how HRV responsiveness could be reduced in order to combat emotional exhaustion in everyday life. Kiecolt-Glaser and colleagues^16 ^suggested a number of interventions for altering stress reactivity, including yoga, meditation, regular exercise, healthy diet, and sleep. Regular mindfulness practices, for example, have already been found to be helpful in this respect^60^. However, our study was carried out using a cross-sectional design that does not allow any conclusions about directionality. Therefore, other pathways of the dynamic interplay between physiological stress reactivity and emotional well-being must also be taken into account. Indeed, there is empirical evidence for reverse or even reciprocal relationships^16^, such that individuals who experience elevated emotional exhaustion also tend to exhibit greater physiological reactivity. Following this causal pathway, it may also be advisable to focus on interventions aimed at fostering well-being, rather than focusing directly on the reactivity itself, in order to minimize the risk of accentuated stress reactivity. Self-induced well-being—for instance, via recalling past situations that still evoke positive emotional states—appears to be promising in this regard^58^.

Despite the numerous strengths of the present study, such as using a novel and ecologically valid method for modeling physiological (HRV) stress reactivity by means of within-person contingencies based on an EMA protocol, some shortcomings must also be acknowledged. First, in comparison with a laboratory study, our field study involved losses in standardization, experimental control, and physiological data quality^4^. Even though we controlled for the most important covariate related to HRV (i.e., physical activity) and we relied on high-quality ECG equipment with additional visual inspection of artifacts, we could not ascertain which other characteristics of the person or situation may have biased the results. Given the conditions in the real-life setting, we were faced with missing (HRV) data and subjects to be excluded, which carries the risk that the missing data may show different HRV values than those in the analysis. Providing many realistic situations in the laboratory may help overcome these shortcomings by providing greater control. Second, the present findings were drawn from pooled subsamples with different sampling schemes. Concerning our sample, it should be noted that most of our subsamples stem from high-stress settings (e.g., police officers in training and health care professionals) and were self-selected (and not sampled randomly) which limits generalizability to the general population^61^. Therefore, future research should examine different randomly selected occupational groups and individuals with various levels of stress and strain in order to replicate the current results. Regarding the mixed sampling protocols, our data included both fixed and random time prompts as well as different retrospective timescales for reporting time pressure of approximately 1–2 hours. In addition, participants were given the option of rejecting the questionnaires or postponing them for up to 45 minutes after receiving the initial prompt. This could have resulted in the fact that some questionnaires were completed when periods of time pressure had already passed, possibly leading to an under-estimation of the relationship with HRV. This sampling variability means that the predictability and timeframe of the questionnaires were different^62^, which is why future studies should investigate whether the results can be replicated when identical procedures are applied. Third, our study included only single proxies for everyday stressors (time pressure), physiological strain indicators (HRV), and emotional outcomes (emotional exhaustion). Therefore, the findings cannot be generalized to other daily hassles (e.g., interpersonal conflicts or much more serious, chronic threats^63^) or to other biomarkers or facets of well-being, which would be a promising avenue for future research. In addition, single-item scales were used to assess time pressure and emotional exhaustion. Single-item scales have been criticized because they are more susceptible to measurement error and are less suitable for the assessment of heterogeneous variables. However, in EMA studies, short instruments are recommended as they are indispensable for maintaining a high compliance^39^, especially in consideration of the already high participant burden due to wearing mobile ECG sensors. Fourth, as noted above, our study was cross-sectional. Therefore, we cannot conclude that stressors evoke accentuated HRV responses and that HRV reactivity produces emotional exhaustion because it could also be the other way around^16^. The underlying causality of these dynamics deserves further investigation in adequate time-lagged or longitudinal designs. Fifth, our investigation specifically referred to momentary, short-term dynamics covering only a few days. A research question worth addressing in future studies would be whether HRV reactivity to stress, operationalized as slopes based on multilevel models, is temporarily stable or whether it is subject to change. It seems plausible that increasing age or past stress and coping experiences, also referred to as stress inoculation and sensitization processes, shape future reactivity and adaptation to upcoming stressors^6,19^. Such a developmental perspective is also provided by the adaptive calibration model with regard to individual differences in trajectories of stress responsivity across life stages^61^. An efficient way to test this hypothesis would be to implement a series of EMAs over time, so-called measurement burst designs^38^. In using such approaches, it would also be interesting to investigate whether the slopes prospectively predict other health outcomes in the long-term (e.g., in burnout^5^) or whether enduring trait personality changes can be observed (e.g., in neuroticism^64^).

To conclude, previous stress reactivity research has examined effects of acute stressors on HRV^11,23,27 ^and investigated effects of psychological or laboratory-based physiological stress reactivity on emotional well-being^3,6,10,18,31^. The present study extends this research by revealing such effects on real-life physiological HRV data and based on contingencies from a within-person perspective. Considering the vast majority of research methods in stress reactivity research that have relied on laboratory settings, psychological self-reports, or one-time measurements^10,18,20^, we highlight the value of the present multimethodological EMA approach, which included mobile ECG recordings. By collecting psychophysiological data across many situations in everyday life and by computing person-specific slopes of stressor-strain relationships, our goal was to provide an innovative, ecologically valid operationalization of HRV reactivity to naturalistic stressors and to investigate its association with emotional well-being in a large adult sample. Research on real-life physiological stress reactivity and its concomitants is still in its infancy, which is why additional studies devoted to examining clear causality as well as investigating different populations and variables are needed to strengthen this evidence. Understanding how everyday stressors impact physiological reactivity and how individual differences in physiological reactivity affect momentary well-being is crucial for deriving practical interventions that can help people avoid health-impairing effects. By utilizing ambulatory, psychophysiological methods, the present study contributes to research on HRV reactivity based on real-life within-person associations and is a first step toward furthering our understanding of dynamic inter- and intraindividual physiological stress processes.

## Data Availability

The anonymized data set and all analysis files are available on the Open Science Framework (OSF; https://osf.io/gwxj3).
